# Alternative Tobacco Product Use and Smoking Quit Attempts Among Teenagers in the United States: A Cross-Sectional Study

**DOI:** 10.7759/cureus.16740

**Published:** 2021-07-29

**Authors:** Colvette Brown, Stanley Nkemjika, Barbara Yankey, Ike Okosun

**Affiliations:** 1 Population Health Sciences, School of Public Health, Georgia State University, Atlanta, USA; 2 Environmental Health, Newton County Health Department, Covington, USA; 3 Public Health/Epidemiology, Georgia State University, Atlanta, USA; 4 Psychiatry and Behavioral Sciences, Interfaith Medical Center, Brooklyn, USA

**Keywords:** tobacco cessation, e-cigarette, alternative tobacco products, adolescents, htps

## Abstract

Background

Public health interventions have heightened awareness of risk factors and ill effects of tobacco use. Though sales of conventional tobacco products have been steadily declining, there is the advent of a new generation of alternative tobacco products marketed with claims of reduced harms and smoking cessation aids. These products are increasing in prevalence and popularity among adolescents.

Aim

The aim of this study is to assess the prevalence of tobacco quit attempts in adolescents in the United States and examine its relationship to the use and self-reported awareness of two alternative tobacco products: e-cigarettes and heated tobacco products (HTPs).

Methods

This is a cross-sectional analysis of data (2,271) from the 2019 National Youth Tobacco Survey (NYTS) of middle and high school students in the United States. Logistic regression analysis was employed to determine the odds of tobacco quit attempts adjusting for age, race, gender, school type, and household tobacco exposure.

Results

The overall prevalence of tobacco quit attempts among e-cigarette users and HTP users was 52.50% and 5.20%, respectively. Results of multivariate regression analyses identified age (OR=0.74, 95% CI:0.57-0.96), race (OR=1.41, 95% CI:1.14-1.75), and household tobacco smoke exposure (OR=1.19, 95% CI:1.01-1.39) as the main factors that are significantly associated with tobacco quit attempts adjusting for all other covariates.

Conclusion

This study did not show a statistically significant association between the awareness and use of e-cigarettes, and heated tobacco products and tobacco smoking quit attempts. Race, age, and exposure to household tobacco smoking were positively associated with quit attempts. Further studies are needed to clarify whether the use and awareness of e-cigarettes and HTPs are associated with tobacco smoking quit attempts within the US adolescent population.

## Introduction

Tobacco smoking has led to over 3 million deaths per year worldwide and 400,000 deaths per year in the US alone [1]. Today, it remains the leading cause of preventable death and disability in the US [2,3]. According to Vangeli et al., the life expectancy for smokers is reduced by three months for every year they continue to smoke over the age of 40 [4]. National anti-tobacco campaigns and other impactful public health interventions have led to increased awareness of tobacco smoking's ill effects [5]. Consequently, there has been a reduction in the sale and prevalence [6,7] of traditional combustible tobacco products (c-cigarette). However, while cigarette sales have declined, there is the emergence of a new generation of tobacco products [8,9]. Marketing expenditures for alternate or non-conventional tobacco products have increased in the past decade [10], which have led to products like electronic cigarettes (e-cigarette) and heated tobacco products (HTPs) increasing prevalence and popularity among smokers [11].

E-cigarettes are a type of electronic nicotine delivery system (ENDS) created to mimic the sensory experience of smoking traditional combustible cigarettes by vaporizing a liquid mixture that consists of propylene glycol, glycerin, flavorings, nicotine, and other chemicals [12]. It is noteworthy that e-cigarettes are commonly marketed with claims of smoking cessation [8] and have become very popular among adolescents. However, nicotine, a component of both the traditional cigarette and e-cigarette, affects adolescents' developing brains and increases the likelihood of addiction [13]. Similarly, there are additional adverse health effects of the toxic impurities in e-cigarette cartridges, for which the full extent of these health effects is still unknown [13].

The burden of disease has been increasing as 3.05 million high school students (19.3% increase) in the United States were current e-cigarette users in 2018 [14,15]. This was an increase from 1.5% in 2011 to 20.8% in 2018. A similar trend was observed among middle school students, with an increase in e-cigarette use from 0.6% in 2011 (60,000 students) to 4.9% (570,000 students) in 2018 [14]. The vast array of e-cigarettes has attracted young people's attention as they are available in various flavors, shapes, and colors [16]. Like e-cigarettes, heated tobacco products (HTPs), also referred to as "heat-not-burn" tobacco products, have been introduced into the US market. Several concerns have been raised about the impact of these non-traditional tobacco products on youth [16]. Like e-cigarettes, HTPs utilize a mechanism whereby heat is used to volatile nicotine to a point below combustion [17], resulting in the production of an aerosol instead of burning, which produces smoke [18]. These products differ in the sense that e-cigarettes heat nicotine in a liquid solution while HTPs heat cigarette-like tobacco sticks. Tobacco companies use persuasive messages that influence the belief that HTPs are more socially acceptable compared to traditional/combustible cigarettes [9]. A study by Marynak et al. revealed that 5.2% of the adult population in the US were aware of heated tobacco products and less than 1% reported ever use of HTPs [19].

Recently, the United States Food and Drug Authority (FDA) authorized the marketing of different HTP products in the country. Thus far, despite the approval and utilization of these products globally, only a few studies have examined the correlations between HTPs and demographic characteristics. Additionally, the impact of electronic cigarettes (e-cigarettes) and heated tobacco products (HTPs) on youth remains a controversial public health problem as it remains unclear whether alternative (non-conventional) products will lead to an uptick in the use of such products or smoking [20].

Within the public health community, there have been arguments about the viability of e-cigarettes to reduce harm or to be used by smokers to supplement or truly substitute conventional tobacco smoking and successfully attain smoking cessation [20,21]. The decision to use e-cigarettes as a smoking cessation intervention among youth remains conflicting; previous studies report inconsistent findings. In 2016, Kalkhoran and Glantz found that the odds of quitting tobacco smoking were 28% lower among e-cigarette users compared to non-e-cigarette users [22]. Another finding from a six-month follow-up study by Pasquereau et al. (2017) revealed that tobacco smokers who concurrently use e-cigarettes are more likely to attempt to quit at least seven times [23]. One study conducted by Kinouani et al. (2017) found that attempts to quit tobacco smoking were reported more by e-cigarette users than any other smoking group [24]. However, results from a comparative study of the differences and similarities of e-cigarette use among adolescents vs. adults showed that among young adults, e-cigarette use was not consistently associated with attempting to quit tobacco smoking [13]. Whether these factors pose a barrier to quitting among youth remains unknown. Based on the literature review, there is a dearth of studies done to examine this disparate phenomenon. Hence, our research will add to the limited body of evidence assessing the relationship between tobacco quit attempts and alternative tobacco product use among the US adolescent population. 

Notably, considering that the use of non-conventional tobacco products is increasing in popularity and prevalence [21] among youth in the US [16], this has significant public health implications. It is therefore imperative that all factors that (1) present a barrier to smoking cessation within this vulnerable population are identified, and (2) examined to implement appropriate, targeted intervention(s) aimed at reducing the prevalence of use and increase the number and success of quit attempts among youth.

Hence, using a cross-sectional design, we aim to: (a) assess the prevalence of tobacco quit attempts among US adolescents in middle and high school and (b) examine the relationship between alternative tobacco product use and awareness to attempts to stop tobacco smoking. Additionally, this study sought to add to a growing body of evidence on youth tobacco-related research and bridge the literature gap.

## Materials and methods

Data source and study population

The National Youth Tobacco Survey (NYTS) is an annual voluntary school-based, self-report cross-sectional survey, conducted by the CDC in collaboration with the FDA, designed to investigate tobacco-related beliefs, attitudes, behaviors, and exposure to pro-and anti-tobacco influences among public middle school (grades 6-8) and high school (grades 9-12) students. The year 2019 was the first time the survey was administered in schools using tablet computers. Participating schools determined whether parental consent would be received actively, whereby parents provided written consent allowing their child to participate in the survey, or passively, whereby parents signed and returned the consent form only if they did not want their child to participate in the survey. Parental consent and respondent assent were obtained for all participants [15].

Sample selection

A three-stage cluster sampling procedure was used to generate a nationally representative sample of U.S. students attending public and private schools in grades 6-12 from all 50 states and the District of Columbia [15]. As defined by the CDC, the primary sampling unit is a county, a group of small counties, or part of a very large county and was selected at random within each stratum. Secondary sampling units, including schools within each selected PSU, were selected randomly within each PSU [25]. At the final sampling stage, classes were selected at random within each school. Student Participation in the 2019 NYTS was voluntary and anonymous, required parental consent and student assent. From the final sample of 325 schools in 2019, 251 participated, yielding a school response rate of 77.2% (or refusal rate of 22.8%) [25]. From a sample of 22,153 students, the total number of student questionnaires completed was 19,018, yielding a student response rate of 85.8% and an overall response rate of 66.3% [25]. Race and ethnicity were separately assessed by self-report with fixed category response options [14]. Students could select one or more of the following categories for the race: American Indian or Alaska Native, Asian, Black or African American, Native Hawaiian or other Pacific Islander, or White. Students could select whether they were Hispanic, Latino, Latina, or of Spanish origin. The National Youth Tobacco Survey (NYTS) was approved by the institutional review board of the US Centers for Disease Control and Prevention (CDC). In 2018, a pilot survey of the NYTS was conducted using two electronic versions, one programmed to align with the paper-based survey and the other to take advantage of electronic administration, including programmed skip patterns and tobacco product images [14]. The number of participants included in the 2019 dataset was 19,018, and there were 421 variables. A detailed description of the 2019 NYTS survey design, questionnaire, and data collection can be found at https://www.cdc.gov/tobacco/data_statistics/surveys/nyts/index.htm [26].

Data collection procedure

The 2019 data collection period spanned from February 15, 2019, to May 24, 2019; during that time, a survey application was used to collect data offline that was loaded onto an electronic tablet [14]. Students absent on the day of survey administration could participate using a web-based version of the questionnaire, which was programmed to mimic the tablet-based application. In addition, skip patterns were programmed in the 2019 questionnaire to reduce respondent burden [14, 15]. This is a secondary analysis of publicly available, de-identified data; therefore, no ethics approval was sought.

Exposure assessment

E-cigarette use was defined as using an electronic cigarette on at least one day in an entire lifetime. Self-reported awareness of e-cigarettes was assessed by the following "Do you believe that e-cigarettes are (less addictive, equally addictive, more addictive) than cigarettes? Options for answers were: Less addictive, equally addictive, more addictive, I have never heard of e-cigarettes, and I don't know enough about these products. Those who chose an option other than "I have never heard of e-cigarettes" were classified as being aware of e-cigarettes. Attempts to quit was defined as selecting (1) or more times to the question: "During the past 12 months, how many times have you stopped using all tobacco products for one day or longer because you were trying to quit all tobacco products for good?" Attempts to quit smoking tobacco products were dichotomized as yes or no based on students' responses to the previous question. Those who selected one or more times were categorized as "yes," and those who chose the answer "I did not try to quit all tobacco products during the past 12 months" were categorized as" no." Students who legitimately skipped the question were excluded. 

Heated tobacco products (HTPs) self-reported awareness and use of HTPs were assessed by the questions respectively "Before today, have you ever heard of heated tobacco products? Have you ever tried a 'heated tobacco product' even just one time?" 

Statistical analysis

The analyses were separately conducted for e-cigarettes and HTPs. In the descriptive statistics, the frequency distribution was reported to describe the characteristics of the population. A bivariate analysis was performed to obtain the crude odds ratio (OR) for examining the relationship between all variables in the study and tobacco quit attempts. Separate multivariate logistic regression analyses were conducted for e-cigarettes and HTPs. Factors associated with tobacco quit attempts were analyzed using logistic regression analyses. Four (4) models were estimated in the multivariate regression analyses for each study's alternative tobacco products. For e-cigarette, in the first model, tobacco quit attempt(s) (the dependent variable) was modeled with socio-demographic factors (age, race, gender, school type, age at initiation of e-cigarette). In the second model, e-cigarette use was added to the model. In the third model, e-cigarette use was substituted with self-reported awareness (of the addictiveness of e-cigarette). In the final model, both e-cigarette use and self-reported awareness of the addictiveness of e-cigarettes were added. The analyses were repeated for HTPs. Results were presented in the form of OR and their confidence intervals (CI). The weight, stratum, and primary sampling units (PSUs) variables provided in the public dataset were incorporated when performing analyses. All statistical data analyses were done using SAS 9.4 statistical software (SAS Institute Inc.Cary, NC).

Description of variables

In this study, the dependent variable was tobacco quit attempt, dichotomized as" yes" or "no." The main independent variables of this study were assessed as binary outcomes, which include e-cigarettes use, Self-reported awareness of the addictiveness of e-cigarette, HTP use, Self-reported awareness of HTPs, and sociodemographic attributes (age, gender, race/ethnicity, school type, initiation age for e-cigarettes and household exposure status).

## Results

A total of 19,018 students participated in the survey. From this sample, 2271 (11.94%) attempted to quit tobacco smoking within the past 12 months, 18,096 (96.75%) had heard of e-cigarettes before and were aware of their addictive nature. A total of 6,356 (33.42%) reported the use of e-cigarettes on at least one day in their lifetime. A total of 2390 (12.57%) and 398 (2.34%) respondents had heard of HTP before and used HTP, respectively. Overall, 52.50% of ever-users of e-cigarettes attempted to quit tobacco smoking within the past 12 months, and the prevalence of HTP use among respondents who attempted to quit tobacco smoking within the past 12 months was 5.20%.

 Description of the sample population

Table 1 shows the socio-demographic characteristics of respondents in the entire study population. Of the total number of respondents (19,018), 50.74% were non-Hispanic White, 24.38% were Hispanic, and 12.33% were Black or African American. The majority of respondents (55.92%) were in high school and between 13-15 years of age (44.42%). The prevalence of household tobacco smoke exposure within the past seven days was 25.28%. Approximately 97% of respondents were aware of the addictive effect of e-cigarettes, and 34.66% had used an e-cigarette on at least one day in their entire life. There was a significantly larger proportion of e-cigarette users (34.7%) compared to HTP users (2.61%), and only 16.52% of respondents had heard of HTPs.

**Table 1:** Characteristics of middle and high school students in the United States, by selected demographic variables - NYTS 2019 Note: Row percent used, unweighted frequency reported. Middle school (Grades 6-8) High School (Grade 9-12). Other †† (American Indian or Alaskan, Asian, Native Hawaiian or other Pacific Islander).

Demographic Characteristics	Frequency	Weighted% (95%CI)
Age in years (n=18,980)		
9-12	3,951	19.200(17.131-21.269)
13-15	8,481	44.420 (42.656-46.185)
16-17	5,050	27.883 (25.437-30.328)
18 years or older	1,498	8.497 (7.463-9.532)
Race/Ethnicity (n=19,018)		
Black/African American	2,288	12.334 (9.674-14.994)
Hispanic	5,564	24.376 (21.312-27.441)
Non-Hispanic White	8,536	50.737 (46.451-55.022 )
Other††	2,630	12.553 (11.055-14.051)
Gender (n=18,902)		
Male	9,803	52.043 (50.393-53. 693)
Female	9,099	47.957 (46.307-49.607)
School Type (n=18,934)		
Middle	8,837	44.081 (39.779-48.383)
High	10,097	55.919 (51.617-60.221)
Household Tobacco smoke exposure (n=18,613)		
Yes	4,586	25.280 (23.336-27.224)
No	14,027	74.720 (72.776-76.664
E-cigarette use (n= 19,018)		
Yes	6356	34.657 (32.536-36.779)
No	12662	65.343 (63.222-67.465)
E-cigarette awareness (n= 18,704)		
Yes	18096	96.993 (96.577-97.408)
No	608	3.008 (2.592-3.423)
HTP use (n= 17,031)		
Yes	398	2.612 (1.641-3.583)
No	16633	97.388 (96.417-98.359)
HTP awareness (n= 14,550)		
Yes	2390	16.520(15.010-18.030)
No	12160	83.480 (81.970-84.990)

Bivariate logistic regression results

Table 2 shows the unadjusted odds of tobacco quit attempts for each study variable using bivariate logistic regression. As compared to respondents who were 13-15 years old, the odds of tobacco quit attempts were highest among adolescents between the ages of 9 and 12 (OR=1.25, 95% CI: 0.897-1.754); this association was not statistically significant. Adolescents within all other age groups had lower odds of attempting to quit smoking compared to those aged 13-15 (see Table 2). Age group 16-17 (OR=0.76, CI: 0.636-0.911) or 18 years or older (OR =0.79, 95% CI: 0.635-0.974) was a protective factor against tobacco quit attempt. Adolescents in the age group 16-17 had 0.76 times lower odds of attempting to quit smoking compared to those in the 13-15 age group (OR=0.76, CI:0.636-0.911). Individuals in the 18 years or older age category had 0.79 times (OR =0.79, CI: 0.635-0.974) lower odds of tobacco smoking quit attempts. Notably, the bivariate analyses showed a statistically significant association between tobacco quit attempts and school type, race, age at initiation of e-cigarette, and household exposure to tobacco smoking. The odds of tobacco quit attempts were 1.36 times (OR= 1.36, CI:1.13-1.63) higher among middle school students compared to high school students. The unadjusted odds of tobacco quit attempts were higher among Hispanics (OR =1.35, CI:1.10-1.66) and Black/African Americans (OR=1.15, CI:0.90-1.46) but lower among other races (OR =0.98, CI: 0.78-1.22) as compared to Non-Hispanic Whites. Adolescents exposed to tobacco smoking in the home (OR=1.25, 95% CI:1.08-1.45), those in middle school (OR = 1.36, 95% CI: 1.13-1.63), those who had their first e-cigarette before age 13 (OR=1.39, 95%CI:1.083-1.772) and females (OR=1.04, 95% CI: 0.895-1.206) were positively linked with higher odds of attempting to quit tobacco smoking. Both e-cigarette use and HTP use were associated with greater odds of attempting to quit tobacco smoking (OR =1.18, CI:0.90-1.54 and OR= 1.07, 95% CI:0.823-1.384 respectively). However, the associations were not statistically significant.

**Table 2:** Bivariate logistic regression examining the relationship between sample characteristics and tobacco quit attempts

Demographic characteristics	Odds Ratio	95% Confidence Interval
9-12 years	1.254	(0.897-1.754)
13-15 years	Reference	Reference
16-17 years	0.761	(0.636-0.911)
18 years or older	0.787	(0.635-0.974)
Race/Ethnicity		
Black/African American	Reference	Reference
Hispanic	1.147	(0.899-1.464)
Non-Hispanic White	1.353	(1.101-1.664)
Other††	0.976	(0.780-1.221)
Gender		
Male	Reference	Reference
Female	1.039	(0.895-1.206)
School Type		
Middle	Reference	Reference
High	1.356	(1.125-1.634)
Smoking characteristics		
Household Tobacco smoke exposure		
No	Reference	Reference
Yes	1.251	(1.076-1.454)
Age at initiation of e-cigarette use		
9-12	1.385	(1.083-1.772)
13-15	1.094	(0.899-1.330)
16-17	Reference	Reference
18 years and older	0.894	(0.710-1.126)
E-cigarette use		
No	Reference	Reference
Yes	1.184	(0.909-1.542)
E-cigarette awareness		
No	Reference	Reference
Yes	0.803	(0.512-1.259)
HTP use		
No	Reference	Reference
Yes	1.067	(0.823-1.384)
HTP awareness		
No	Reference	Reference
Yes	1.079	(0.902-1.290)

Multivariate logistic regression results for e-cigarettes

Table 3 shows the frequency of tobacco quit attempts and the corresponding adjusted odds ratio (aOR) for all the study variables, except for HTP use and HTP awareness (assessed separately). The aORs were estimated using four different multivariate logistic regression models. Model 1 shows the aORs for the sociodemographic variables age, race, gender, school type, household exposure to tobacco smoking, and age at initiation of e-cigarette. After adjusting for all covariates, there was no longer a significant association between school type and tobacco quit attempts (aOR= 1.08, 95% CI:0.811-1.439). The adjusted estimates show that age, race, and household exposure to tobacco smoking were significantly associated with attempting to quit tobacco smoking. Adolescents within the 16-17 age group were 0.75 times (aOR=0.75, 95% CI: 0.578-0.972) less likely to attempt to quit tobacco smoking compared to those within the 12-15 age group. Conversely, Hispanic Whites were 1.38 times (aOR=1.38, 95% CI:1.110-1.711) as likely to attempt to quit tobacco smoking when compared with non-Hispanic Whites after adjusting for age, gender, school type, household exposure to tobacco smoking, and age at initiation of e-cigarette. The odds of tobacco quit attempt were 1.19 times (aOR=1.19, 95% CI:1.1190-1.390) as likely among adolescents who are exposed to tobacco smoking in the home when compared to those without household tobacco smoke exposure after adjusting for age, race, gender, school type, and age at initiation of e-cigarette. All sociodemographic variables in Model 1 (age, race, gender, school type, household exposure to tobacco smoking, and age at initiation of e-cigarette) were adjusted for in subsequent models. In Model 2, e-cigarette use was added to the model to examine its relationship to a tobacco quit attempt. The adjusted estimates also showed that age, race, and household tobacco exposure were associated with a tobacco quit attempt. Hispanics were 1.39 times (aOR=1.388, 95% CI:1.118-1.722) as likely to attempt to quit tobacco smoking compared to non-Hispanic Whites, after adjusting for age, race, gender, school type, household exposure to tobacco smoking, and age at initiation of e-cigarette. Similarly, the odds of tobacco quit attempts were 1.19 (95% CI: 1.013-1.390) times as likely among adolescents exposed to tobacco smoking in their homes compared to those without household tobacco smoke exposure. See Table 3 for more results. 

**Table 3:** Prevalence of tobacco quit attempt by socio-demographic characteristics, and multivariate association of the use and awareness of e-cigarette and smoking quit attempts Note:  --- Variable not included in the model, Middle school (grade 6-8), High school (Grade 9-12),  NH-White (Non-Hispanic white), Black/AA (Black or African American), other (Asian, Pacific Islander, Hawaiian native, American Indian).

Participant characteristics	Tobacco quit attempt N (%)		Quit attempt OR (95% CI)			
	Yes	No	Model 1	Model 2	Model 3	Model 4
Age						
9-12	174 (3.890)	91 (2.019)	1.196 (0.834-1.715)	1.190 (0.829-1.708)	1.226 (0.863-1.740)	1.211 (0.850-1.726)
13-15	977(23.939	645 (15.586)	Reference	Reference	Reference	Reference
16-17	816 (21.158)	704 (18.097	0.749(0.578-0.972)	0.746 (0.575-0.969)	0.742 (0.573-0.961	0.738 (0.569-0.958)
18 years or older	302 (8.378)	261 (6.934)	0.842 (0.591-1.200)	0.839 (0.588-1.196)	0.823 (0.577-1.176)	0.820(0.573-1.171)
Race/Ethnicity						
NH-White	1111 (31.496)	908 (25.366)	Reference	Reference	Reference	Reference
Black/AA	243(6.099)	170 (4.283)	1.170 (0.860-1.593)	1.171(0.860-1.593)	1.156 (0.849-1.574)	1.154 (0.848-1.572)
Hispanic	672 (14.468)	431 (8.610)	1.378 (1.110-1.711)	1.388 (1.118-1.722)	1.402 (1.128-1.743)	1.411 (1.136-1.753)
Other	245 (5.303)	193 (4.374)	0.978 (0.745-1.285)	0.992 (0.755-1.305)	0.980 (0.747-1.287)	0.994 (0.756-1.308)
Gender						
Male	1239 (30.093)	921 (22.826)	Reference	Reference	Reference	Reference
Female	1019 (26.984)	774(19.702)	1.078 (0.916-1.268)	1.078 (0.915-1.269)	1.070 (0.911-1.257)	1.070 (0.910-1.258)
School Type						
High	1609 (42.354)	1318 (33.894)	Reference	Reference	Reference	Reference
Middle	649 (14.937)	379 (8.816)	1.081 (0.811-1.439)	1.091 (0.818-1.455)	1.060 (0.796-1.412)	1.070 (0.803-1.427)
Household tobacco smoke exposure						
No	1257 (32.452)	1049 (26.662)	Reference	Reference	Reference	Reference
Yes	953 (24.676)	623 (16.210)	1.081 (0.811-1.439	1.187 (1.013-1.392)	1.187 (1.015-1.388)	1.185 (1.012-1.387)
Age at initiation of e-cigarette						
9-12	457 (12.376)	269 (7.12)	1.038 (0.757-1.421)	1.032 (0.752-1.415)	1.014 (0.735-1.399)	1.008 (0.729-1.393)
13-15	725 (19.560)	519 (14.25)	0.900 (0.724-1.119)	0.899 (0.724-1.118)	0.893 (0.714-1.118)	0.893 (0.714-1.117)
16-17	673 (19.574)	554 (15.59)	Reference	Reference	Reference	Reference
18 years or older	211 (6.096)	189 (5.431)	0.883 (0.651-1.197	0.882 (0.650-1.197)	0.880 (0.648-1.195)	0.879 (0.647-1.195)
E-cigarette use						
No	1528 (38.419)	39 (0.881)	------	Reference	------	Reference
Yes	1529 (38.419)	174 (4.215)		2.609 (0.918-7.415)		2.594 (0.856-7.866)
E-cigarette awareness						
No	55 (1.4623)	39 (0.881)	------	------	Reference	Reference
Yes	2163 (55.775)	1058 (41.881)			0.993 (0.918-7.223)	0.789 (0.468-1.333)

Model 3 showed the adjusted odds ratios when e-cigarette use was removed from the model and replaced with e-cigarette awareness. The adjusted estimates show that age, race, and exposure to tobacco smoking in the home are significantly associated with tobacco quit attempts. After adjusting for age, race, gender, school type, household exposure to tobacco smoking, and age at initiation of e-cigarette, the likelihood of tobacco quit attempt was 1.40 times (95% CI:1.128-1.743) as likely among adolescents who are Hispanic Whites compared to non-Hispanic Whites. The odds of tobacco quit attempts were 1.19 times (95% CI: 1.015-1.388), as likely among adolescents living in a household with tobacco smoke exposure. There was a statistically significant association between the age group 16-17 years, and tobacco quit attempts (aOR = 0.742, 95% CI:0.573-0.961). Adolescents between 16 and 17 years old were less likely to quit tobacco smoking when compared to those who are 13-15 years (See Table 2).

Model 4 showed the adjusted odds ratios for all the study variables when e-cigarette use and e-cigarette awareness were added. After adjusting for all other variables, the odds of attempting to quit tobacco smoking were 0.74 times (aOR=0.738, 95% CI:0.569-0.958) as likely among adolescents between 16 and 17 years of age compared to those aged 13-15. Similarly, race and household tobacco smoke exposure were associated with tobacco quit attempts; this was observed across all the models. Compared to non-Hispanic whites, the odds of tobacco quit attempts were 1.41 (95% CI:1.136-1.753) as likely among Hispanics and 1.19 times (95% CI:1.012-1.387) as likely among adolescents who are exposed to tobacco smoking in the home. Middle school students (aOR=1.07, 95% CI: 0.80, 1.43), females (aOR=1.07, 95% CI:0.910-1.258), and those who initiated e-cigarette use between the ages of 9-12 years (aOR=1.01, 95% CI:0.729-1.390) had a higher odds ratio for attempting to quit tobacco smoking when compared to high school students, males and those who initiated e-cigarette use between the ages of 16-17 respectively. While the odds for attempting to quit smoking were 2.59 times (95% CI: 0.86-7.87) as likely among e-cigarette users, it was not statistically significant; this lack of statistical significance was consistent in all the previous models.

Multivariate logistic regression results for HTPs

Table 4 shows the frequency of tobacco quit attempts and the corresponding adjusted odds ratio (aOR) for all the study variables, excluding e-cigarette use and awareness. Four different multivariate logistic regression models were created to examine the relationship between tobacco quit attempts and the use and awareness HTPs.

**Table 4:** Prevalence of tobacco quit attempt by socio-demographic characteristics and multivariate association of the use and awareness of HTPs and tobacco smoking quit attempts Note:  --- Variable not included in the model, Middle school (grade 6-8), High school (Grade 9-12), NH-White (Non-Hispanic white), Black/AA (Black or African American), other (Asian, Pacific Islander, Hawaiian native, American Indian).

Participant Characteristics	Tobacco quit attempt N (%)		Quit attempt OR (95% CI)			
	Yes	No	Model 1	Model 2	Model 3	Model 4
Age						
9-12	174 (3.890)	91 (2.019)	1.168 (0.839-1.626)	1.510 (1.010-2.258)	1.468 (1.033-2.087)	1.514 (1.008-2.273)
13-15	977(23.939)	645 (15.586)	Reference	Reference	Reference	Reference
16-17	816 (21.158)	704 (18.097)	0.795 (0.632-1.000)	0.766 (0.591-0.993)	0.830 (0.643-1.072)	0.804 (0.614-1.052)
18 years or older	302 (8.378)	261 (6.934)	0.811 (0.627-1.048)	0.783 (0.596-1.028)	0.824 (0.625-1.086)	0.807 (0.604-1.078)
Race/Ethnicity						
NH-White	1111 (31.496)	908 (25.366)	Reference	Reference	Reference	Reference
Black/AA	243(6.099)	170 (4.283)	1.099 (0.858-1.408)	1.190 (0.854-1.658)	1.252 (0.870-1.802)	1.242 (0.849-1.817)
Hispanic	672 (14.468)	431 (8.610)	1.282 (1.035-1.589)	1.319 (1.045-1.664)	1.249 (0.966-1.614)	1.310 (1.015-1.690)
Other	245 (5.303)	193 (4.374)	0.962 (0.754-1.227)	0.891 (0.709-1.119)	0.952 (0.747-1.214)	0.882(0.696-1.117)
Gender						
Male	1239 (30.093) 921(22.826)	921(22.826)	Reference	Reference	Reference	Reference
Female	1019 (26.984) 774(19.702)	774 (19.702)	1.078 (0.916-1.268)	1.078 (0.915-1.269)	1.070 (0.911-1.257)	1.070 (0.910-1.258)
School type						
High	1609 ( 42.354)	1318 (33.894)	Reference	Reference	Reference	Reference
Middle	649 (14.937)	379 (8.816)	1.068 (0.823-1.387)	1.064 (0.790-1.437)	1.083(0.795-1.473)	1.098 (0.784-1.538)
Household tobacco smoke exposure						
No	1257 (32.452)	1049(26.662)	Reference	Reference	Reference	Reference
Yes	953 (24.676)	623(16.210)	1.231 (1.056-1.434)	1.226 (1.032-1.456)	1.200 (1.003-1.436)	1.228 (1.027-1.468)
HTP Use						
No	1694 (52.122)	128(39.033)	------	Reference	------	Reference
Yes	165 (5.198)	117(3.647)		0.948 (0.719-1.251)		0.908 (0.599-1.377)
HTP awareness						
No	1367 (45.693)	1058(34.774)	------	------	Reference	Reference
Yes	356 (11.455)	254 (8.078)			1.007(0.818-1.239)	1.001 (0.717-1.397)

Model 1 shows the aORs for the sociodemographic variables age, race, gender, school type, and household exposure to tobacco smoking. After adjusting for all the other variables, there was no longer a significant association between students within the 9-12 age group (aOR=1.168, 95% CI:0.839-1.626) and tobacco quit attempts. Similarly, after adjusting for age, race, gender, and household tobacco smoke exposure, there was no longer a statistically significant association between school type (aOR =1.068, 95% CI:0.823-1.387) and tobacco quit attempts. Hispanic Whites were 1.28 times (aOR=1.282, 95% CI: 1.035-1.589) as likely as their counterparts to make an attempt to quit tobacco smoking (non-Hispanic White) after adjusting for age, gender, school type, and household exposure to tobacco smoking. The odds of tobacco quit attempt was 1.21 times (aOR= 1.231, 95% CI:1.056-1.434) as likely among adolescents who are exposed to tobacco smoking in the home when compared to those without household tobacco smoke exposure, after adjusting for age, race, gender, school type, and age at initiation of e-cigarettes.

Model 2, HTP use, was added to the model to examine its relationship to tobacco quit attempts. The adjusted estimates show that adolescents aged 9-12 are 1.51 times (95% CI:1.010-2.258) as likely to quit tobacco smoking when compared to those who are 13-15 years old. Conversely, adolescents aged 16-17 had 0.77 times (95% CI:0.591-0.993) the odds (aOR= 0.766, 95% 0.591-0.993) of tobacco quit attempt compared to those in the 13-15 age group. After adjusting for all other variables in the model, the odds of tobacco quit attempts were 1.32 times (95% CI:1.045-1.664) as likely among Hispanic Whites compared to non-Hispanic Whites. Exposure to tobacco smoking in the home was another significant factor of tobacco quit attempts. Adolescents who live in a household where they are exposed to tobacco smoking were 1.23 times (aOR=1.226, 95% CI:1.032-1.456) as likely to attempt to quit tobacco smoking compared to those unexposed to tobacco smoking in the home.

In Model 3, HTP use was replaced with HTP awareness. After adjusting for all covariates, the odds of tobacco quit attempt were 1.47 times (aOR =1.468, 95% CI:1.033-2.087) as likely among those between the ages of 9-12 compared to those who are 13-15 years old. Notably, the odds of tobacco quit attempts decreased with increasing age when HTP awareness was added to the model. The aOR for adolescents between 16-17 years old was 0.830, 95% CI:0.643-1.072 and for those 18 years or older, aOR = 0.824 (95% CI:0.625-1.086). There was a statistically significant association between household tobacco smoke exposure and tobacco quit attempts. The odds of tobacco quit attempts were 1.20 times (95% CI: 1.003-1.436) as likely among adolescents living in a household with tobacco smoke exposure. There was no statistically significant association between HTP awareness (aOR=1.007, 95% CI: 0.818-1.239) and tobacco quit attempts.

Both HTP use and HTP awareness were included in the final model. The estimated odds ratio for making a quit attempt decreased with increasing age after adjusting for age, race, gender, school type, and household tobacco exposure. After adjusting for other variables, the odds of tobacco quit attempt were 1.51 times (aOR= 1.514, 95% CI:1.008-2.273) as likely among adolescents aged 9-12 compared to those aged 13-15. Hispanic adolescents were more likely to have made a quit attempt compared to non-Hispanic White adolescents (aOR=1.310, 95% CI:1.015-1.690) after adjusting for all other variables. Similarly, adolescents exposed to tobacco smoke in the home were 1.23 times (95% CI:1.027-1.468) as likely to attempt to quit tobacco smoking compared to their peers who are unexposed to tobacco smoking in the home. There was no statistically significant association between HTP use or awareness, and tobacco quit attempt (aOR= 0.91; 95% CI:0.599-1.377) and (aOR=1.00; 95% CI: 0:717-1.397) respectively.

Table 5 shows the unadjusted and adjusted odds of tobacco quit attempts among users of both HTPs and e-cigarettes. As compared to non-HTP users, the odds of smoking quit attempts were 0.88 times (95% CI: 0.572-1.351) as likely among HTP users after adjusting for age, race, gender, school type, e-cigarette awareness (addictive property), and household exposure to tobacco smoking which was not statistically significant. Conversely, the odds of tobacco quit attempts were 1.17 times (95% CI: 0.869-1.581) as likely among e-cigarette users compared to non-e-cigarette users after adjusting for all other covariates, which were also not statistically significant. 

**Table 5:** Unadjusted and adjusted OR for the relationship between the combined use of e-cigarettes and HTPs and tobacco quit attempts ††Adjusted for age, race, gender, school type, e-cigarette awareness, and household exposure to tobacco smoking. OR: Odds ratio

Characteristics	Unadjusted OR (95% CI)	Adjusted OR (95% CI)	P-value
HTP use ††			
Yes	1.067 (0.727-1.351)	0.879 (0.572-1.351)	0.55
No	Reference	Reference	
E-cigarette use††			
Yes	1.184 (0.909-1.542)	1.172 (0.869-1.581)	0.29
No	Reference	Reference	

## Discussion

Based on the results of our study, there is an overall higher prevalence of tobacco quit attempts among adolescents between 13-15 years old (23.9%), non-Hispanic Whites (31.5%), males (30.1%), high school students (42.4%), and among e-cigarette users (52.5%). Notably, a higher proportion of e-cigarette users attempted to quit smoking than their non-e-cigarette-using counterparts (52.5% vs. 38.4%). This finding is consistent with results from a study conducted in Korea by Kang and Cho (2019), which investigated the prevalence and association of HTP use [27].

We hypothesized that the use and awareness of e-cigarettes and HTPs were strongly associated with a tobacco quit attempt. However, despite numerous advertising and media messages claiming e-cigarettes to be a safer alternative to c-cigarettes and as a smoking cessation aid, findings from our study showed no statistically significant association between e-cigarette use and tobacco quit attempts among adolescents in the US, after adjusting for all covariates. This finding was consistent with studies done in Finland [8], Canada [11], and the US [13,28,29], which found no association between e-cigarette use and tobacco smoking cessation among adolescents and adults. Similarly, our analyses based on gender stratification (result not shown) showed no association between e-cigarette use and tobacco quit attempts (aOR= 1.01, 95% CI: 0.70-1.463, P= 0.9518). Additionally, our study showed no statistical significance between the self-reported awareness of the addictiveness of e-cigarettes and tobacco quit attempts within the past year. This finding was also consistent with a 2014 study conducted in the US, which reported no significant association between e-cigarette awareness and past-year quit attempts [20]. However, our study's findings conflicted with the results of a longitudinal study that revealed that e-cigarette use for cessation was associated with increased odds of smoking cessation at six and 12 months follow-up compared to non-users of e-cigarettes [23]. 

A possible explanation for that finding may stem from the influence of the social and physical environment on one's health decisions and behaviors. The majority of participants in this study noted that the two main reasons for their use of e-cigarettes were: (a) they were being used by a friend or family member, (b) they were curious about them. It is, therefore, possible that adolescents may have begun using these products because they are trendy, popular, publicly available [11], or as a consequence of the influence of peers, family members, and advertisements (a risk factor for use) and not to aid in tobacco quit attempts [15]. Also, the ability to experiment with the nicotine content in electronic cigarettes may increase the likelihood of addiction, leading to increased use but no benefit of smoking cessation. In 2013, Sutfin et al. found that 12% of e-cigarette users had never smoked a conventional cigarette or any other tobacco product. Sutfin et al.'s study may indicate that adolescents use e-cigarettes to substitute for traditional tobacco (combustible cigarette) rather than as a smoking cessation aid [28]. Another explanation for the lack of association may be a consequence of the cross-sectional study design. There may not have been an adequate amount of time in between adolescents becoming aware of the addictive properties of e-cigarettes and their decision to quit tobacco smoking [13]. Although this study did not examine poly-tobacco use, research shows that it is more difficult for tobacco smokers to quit smoking when using alternative tobacco products and multiple conventional tobacco products concurrently [30]. Therefore, the possibility exists that e-cigarette users in this study also used various tobacco products, making it more difficult for them to quit. Also, how e-cigarette use (one of the independent variables) was defined differs between studies. In this study, e-cigarette use was defined as using an e-cigarette on at least one day in the entire lifetime. In contrast, e-cigarette use was defined by Pasquereau et al. (2017) as regular use of e-cigarettes within the past 30 days [23].

Our study also showed no significant association between tobacco quit attempts with the use and awareness of HTPs, respectively. This finding was consistent with the literature on studies done in both US and abroad, demonstrating no correlation between HTP use and awareness with tobacco quit attempts (CHOICE-STRATA) [23]. Our study suggests that age, race, and household exposure to tobacco smoking were significantly associated with conventional tobacco smoking quit attempts with regards to sociodemographic characteristics. Additionally, the burden of evidence shows that there are disparities among racial/ethnic groups in relation to smoking behaviors, such as quit attempts [6,31]. Our study supports this evidence as there were notable differences in tobacco quit attempts among different racial/ethnic groups. Hispanic Whites were more likely to attempt quitting tobacco smoking when both e-cigarette use and awareness and HTP use and awareness were examined in our analysis (OR=1.41, 95% CI:1.14-1.75 and OR=1.31, 95% CI:1.02-1.36 respectively). This finding was consistent with a 2011 study that reported significant quit attempts correlates with race/ethnic group, which found that Hispanics were more likely to have made tobacco quit attempts than Whites [6,30,31,32].

Our study also showed a positive association between tobacco quit attempts and household exposure to tobacco smoking. This finding supports a similar result from several studies conducted in 14 countries [33], as exposure to smoking in the home was positively associated with attempting smoking cessation. The ecological model of health behavior could explain this result in social and environmental interactions, which can influence smoking behaviors among adolescents [22]. Abrantes et al. (2009) reported that a generalized belief of tobacco's adverse health-related sequelae is related to attempting to quit. Adolescents in this study exposed to tobacco smoking at home may have observed firsthand the negative implications of smoking on one's health [5]. They may have had to become caregivers to family members who have become disabled due to tobacco smoking, or they may have lost a family member from illnesses associated with tobacco use.

Another finding from our study showed an overall lower odds ratio for tobacco quit attempts for older adolescents compared to younger adolescents; this is consistent with the results of a study by Abrantes et al. 2009 [5]. Biological nicotine naivety among younger smokers has been attributed to this finding [34]. We also demonstrated that e-cigarettes (34.7%) are more prevalent among middle and high school students than HTPs (2.6%). This result was similar to findings from studies conducted in Japan [34], Korea [27], and the US [32]. Notably, HTP use in Japan had increased tenfold within two years of introduction to the Japanese market [34]. Evidence in the literature suggests that HTP was projected to surpass e-cigarettes, which had increased 20-fold within three years of introduction in the Korean market [27]. Therefore, it is imperative that though HTP prevalence among adolescents is presently lower than e-cigarettes in the US, there needs to be monitoring and surveillance of this product, particularly within the adolescent population, to identify use patterns and to implement fast and proactive control measures to prevent epidemic rates. While there was no statistical significance between the use and awareness of HTP or the use and awareness of e-cigarettes and smoking, quit attempt, a biological/clinically significant association may exist. 

The marketing claims of tobacco companies regarding the benefit of alternative tobacco products such as smoking cessation aid remain controversial [35]. A literature review shows a lack of consistency about the positive association between tobacco smoking quit attempts and the use of alternative tobacco products. A previous study by Kalkhoran and Glantz from 2016 found that the odds of discontinuing tobacco smoking were 28% lower among e-cigarette users compared to non-e-cigarette users [22]. Similarly, the results of a comparative study by Carroll Chapman and Wu (2014) showed that e-cigarette use was not consistently associated with a tobacco quit attempt [13]. Studies in support of the lack of association between e-cigarette use and tobacco quit attempts include studies done in Finland [8], Canada [17], and the US [36]. However, Kinouani et al. (2017) reported that smoking quit attempts were informed more by e-cigarette users than any other smoking group [24]. Also, results from a six-month follow-up study revealed that e-cigarette users had a higher likelihood of attempting to quit smoking at least seven times than non-e-cigarette users [28].

Strengths and limitations

There are some strengths and weaknesses of this study that may have implications in interpreting the results. The strengths of this study are (1) the data is from the National Youth Tobacco Survey, a nationally representative sample of middle and high school students in the US, hence representative in scope; (2) the questionnaires and measurements in the National Youth Tobacco Survey were done using excellent techniques. The training and quality control measures of the National Youth Tobacco Survey give added reliability to the data and the results of this study; (3) it provides an understanding of the prevalence of tobacco quit attempts by sociodemographic characteristics among middle and high school students in the US. 

There are some limitations of this study that need to be noted. The data was cross-sectional, allowing no causal conclusions since it is not known whether the factors that were examined preceded the outcome. In this study, respondents provided information that might be subject to reporting bias; thus, the true prevalence of tobacco quit attempts may be under-reported. This study also did not consider the possibility of concurrent use of other cessation aids such as nicotine patches, sprays, etc. Future studies to compare the prevalence of quit attempts between individuals who are solely using alternative tobacco products as cessation aids and individuals using a combination therapy should be conducted to provide further insight into the factors associated with a quit attempt.

Public health practice and policy implications

The results of this study highlight the importance of comprehensive, efficacious public health interventions. A strong focus should be placed on tailoring interventions to the adolescent population, including health education and promotion geared towards increasing awareness of the deleterious effects of alternative tobacco product use. Tobacco health information must be communicated in plain language and should consider the health literacy level of the target population; this allows the message to be easily understood and interpreted. School-based interventions may play a critical role in reducing the appeal and acceptability of alternative tobacco products. One recommendation for intervention is to incorporate tobacco and substance use education into the school curriculum. This will provide an avenue through which adolescents will become more knowledgeable about the implications of tobacco use, thereby allowing them to make more informed decisions about the use of tobacco products.

Additionally, providing free tobacco cessation counseling at school and incentivizing students to participate in interscholastic competitions to deter tobacco product use and promote tobacco quit attempts may prove effective at mitigating the risks associated with tobacco use. At the national level, implementing strategies to reduce the availability and marketing of alternative tobacco products should be considered. One approach that can be taken to reduce the prevalence and use among adolescents is to increase the taxes and sales price of these products, making them less affordable for adolescents. In addition, healthcare providers may implement tobacco screening for adolescents in the clinical setting to assess tobacco use behaviors and provide necessary resources to successfully achieve smoking cessation [35]. Given that the result of this study revealed that race/ethnicity was associated with tobacco quit attempts, it is imperative to implement racial-ethnic specific culturally tailored interventions to increase self-efficacy and belief about the health-related consequences of tobacco use. Such an intervention may lead to higher rates of quitting.

## Conclusions

This study showed no association between conventional tobacco quit attempts with the awareness and use of e-cigarettes and HTP. However, race, age, and exposure to household tobacco smoking were positively associated with a quit attempt. The lack of association between the use and awareness of e-cigarette and HTPs warrants the need for a more robust prospective study to determine the true nature of the relationship between use and awareness of alternative tobacco products and quit attempts. Future studies using a prospective study design may provide more useful information about quit attempts and explore the differences in successful quit attempts among different sociodemographic groups within the U.S. adolescent population. Finally, future interventional approaches may consider parental or guardians' knowledge of e-cigarettes and HTPs, which may serve as a foundation to advocate for changes to national e-cigarette and HTP regulation policies.
